# Rotational-motion measurement of the sacroiliac joint using upright MRI scanning and intensity-based registration: is there a sex difference?

**DOI:** 10.1007/s11548-022-02806-w

**Published:** 2022-12-24

**Authors:** Tetsuro Tani, Masaki Takao, Mazen Soufi, Yoshito Otake, Norio Fukuda, Hidetoshi Hamada, Keisuke Uemura, Yoshinobu Sato, Nobuhiko Sugano

**Affiliations:** 1 Department of Orthopaedic Medical Engineering, Osaka University Graduate School of Medicine, Suita, Osaka Japan; 2 Department of Orthopaedic Surgery, Osaka Police Hospital, Osaka, Japan; 3 Department of Orthopaedic Surgery, Osaka University Graduate School of Medicine, 2–2 Yamadaoka, Suita, Osaka Japan; 4 Graduate School of Science and Technology, Nara Institute of Science and Technology, Ikoma, Nara Japan; 5 Center for Information and Neural Networks (CiNet), Advanced ICT Research Institute, National Institute of Information and Communications Technology (NICT), Suita, Osaka Japan

**Keywords:** Sacroiliac joint, Upright MRI, Sex difference

## Abstract

**Purpose:**

The sacroiliac joint (SIJ) has attracted increasing attention as a source of low back and groin pain, but the kinematics of SIJ against standing load and its sex difference remain unclear due to the difficulty of in vivo load study. An upright magnetic resonance imaging (MRI) system can provide in vivo imaging both in the supine and standing positions. The reliability of the mobility of SIJ against the standing load was evaluated and its sex difference was examined in healthy young volunteers using an upright MRI.

**Method:**

Static (reliability) and kinematic studies were performed. In the static study, a dry bone of pelvic ring embedded in gel form and frozen in the plastic box was used. In the kinematic study, 19 volunteers (10 males, 9 females) with a mean age of 23.9 years were included. The ilium positions for the sacrum in supine and standing positions were measured against the pelvic coordinates to evaluate the mobility of the SIJ.

**Results:**

In the static study, the residual error of the rotation of the SIJ study was < 0.2°. In the kinematic study, the mean values of SIJ sagittal rotation from supine to standing position in males and females were − 0.9° ± 0.7° (mean ± standard deviation) and − 1.7° ± 0.8°, respectively. The sex difference was statistically significant (*p* = 0.04). The sagittal rotation of the SIJ showed a significant correlation with the sacral slope.

**Conclusion:**

The residual error for measuring the SIJ rotation using the upright MRI was < 0.2°. The young healthy participants showed sex differences in the sagittal rotation of the SIJ against the standing load and the females showed a larger posterior rotation of the ilium against the sacrum from the supine to standing position than the males. Therefore, upright MRI is useful to investigate SIJ motion.

## Introduction

The sacroiliac joint (SIJ) consists of the sacrum and pelvis, connects the spine to the pelvis, and transfers the axial load between the spine and lower legs [1]. The SIJ has attracted increasing attention as a source of low back and groin pain [2]. Recent studies have reported a higher prevalence of low back pain due to the SIJ, with some reports estimating that the SIJ is the actual source of pain in 15–30% of cases [3–5]. While the mechanism of SIJ-related low back pain was not clarified, it has been reported that hypermobility or laxity of SIJ was one of the causes [6, 7]. Indeed, a previous report has showed that fixation of SIJ and SIJ motion was performed as a treatment for low back pain [7]. SIJ pain and low back pain have been reported more frequently in females than in males [8–10], although the sex difference in the mobility of the SIJ has not been well studied. Although some studies have reported SIJ motion using finite element studies [11–13], few studies have been performed in vivo. This is because it is difficult to measure the mobility of the SIJ against the standing load on plain radiographs due to the difficulty in matching the radiographic parameters between supine and standing position. While a previous study using computed tomography (CT) investigated SIJ motion between trunk flexion and trunk extension by voxel-based registration [14], the use of radiation carried risks and there was a lack of data for healthy females. An upright magnetic resonance imaging (MRI) system can provide in vivo imaging in the supine and standing positions using intensity-based registration and it is possible to quantify the mobility of the SIJ against the standing load without the need for radiation. The investigation of SIJ motion in standing load between supine and standing is important to elucidate the pathology of SIJ pain. The aims of this study were two-fold: (1) to evaluate the reliability of measurements of SIJ mobility using the upright MRI; and (2) to clarify the sex difference in the mobility of the SIJ against the standing load.

## Methods

### Static (reliability) study

**Fig. 1:**
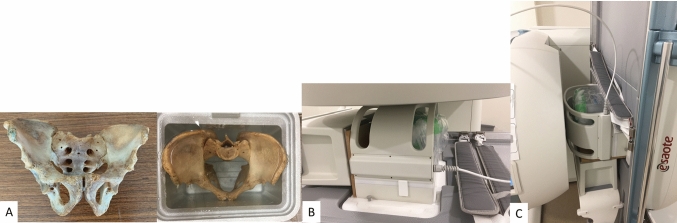
**A** A dry bone of the pelvic ring was embedded in gel foam and frozen in a plastic box. A dry bone of the pelvic ring in the simulated supine **B** and standing position **C**

To assess the reliability of the rotation measurements, the rotations were first investigated in a static dry bone of pelvic ring embedded in gel foam and frozen in a plastic box (Fig. 1). The pubic symphysis and bilateral SIJ were fixed using a glue. The left SIJ was scanned in the simulated supine and standing position using a three-dimensional (3D)-upright MRI system (0.25 Tesla; E-MRI Brio G-Scan, Esaote Spa, Italy) (Fig. 1). A 3D-hybrid contrast-enhanced gradient-echo sequence (3D HYCE) with a 300 mm × 300 mm × 115 mm field of view was used. The repetition and echo times were set to 8 ms and 4 ms, respectively. The images were acquired in the axial direction, and the geometrical distortion correction was activated in the workstation system (ver.4.14.00 F050101) to cope with the magnetic field inhomogeneity. The geometrical distortion correction in this version was investigated using plastic block phantom. The phantom was immersed in water and imaged without and with geometrical distortion correction. The phantom was extracted from the images by thresholding the pixel values. The effect of the distortion correction performance was assessed by calculating the average inter-surface error (i.e., distance) between the extracted phantom and correct phantoms at different slices within the whole field of view (FOV). The error without and with geometrical distortion correction were 1.07 mm and 0.85 mm, respectively.

**Fig. 2:**
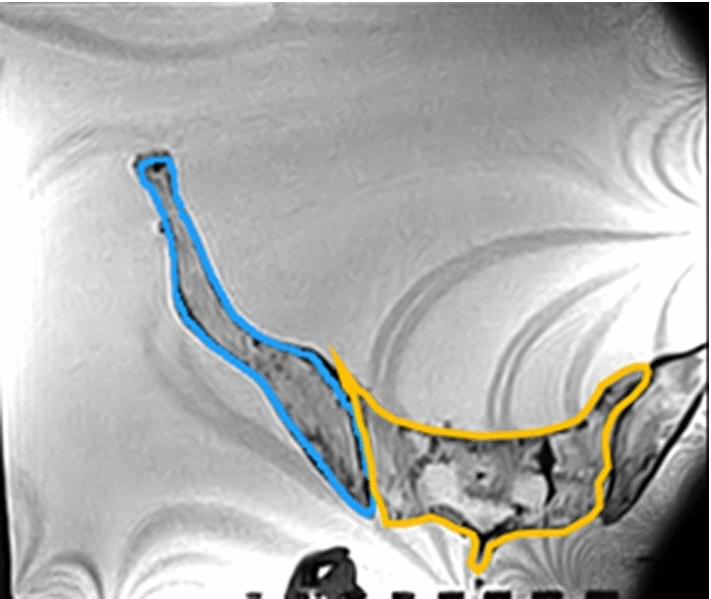
The labels of the sacrum and ilium on axial view of MRI. Blue line and orange line indicate left ilium and sacrum, respectively

The rotation angles were estimated based on intensity-based image registration. The labels of the sacrum and ilium on axial view of MRI were traced manually to restrict the registration on those regions (Fig. 2). A two-step intensity-based volumetric image registration method of the sacrum or ilium between the supine and standing MRI was performed. As SIJ rotation, axial rotation of the ilium relative to the sacrum was calculated. First, the sacrum regions were registered, and the estimated transformation was used to initialize the second transformation that was based on the ilium regions. The rotation angles of the SIJ were calculated from ilium transformation parameters. A mutual information-based objective function was used in optimizing the transformation parameters. The image registration was performed using an open-source tool (Elastix, ver. 4.9) [15].

**Fig. 3:**
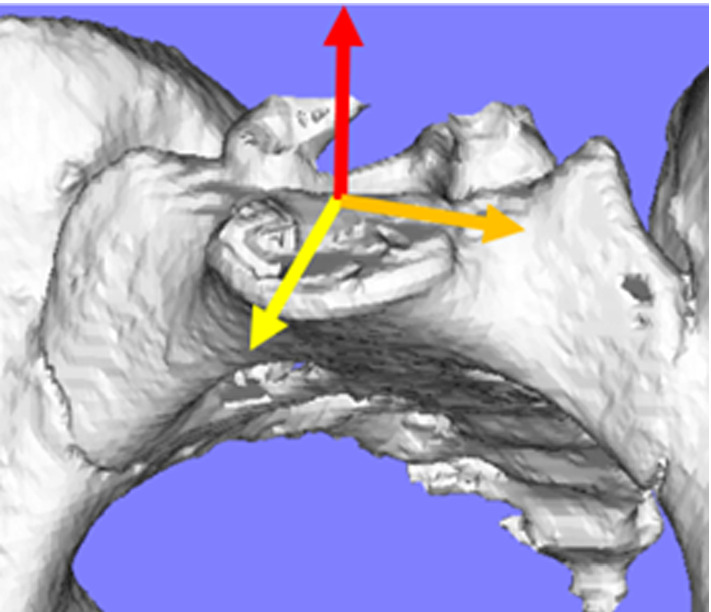
A reference coordinate system was defined on the basis of the shape of the sacral upper vertebra. Each line showed x-axis (yellow line), y-axis (orange line) and Z-axis (red line)

A reference coordinate system was defined based on the basis of the shape of the sacral upper vertebra as follows. A y-axis was set to be the line connecting the anterior and posterior borders of the endplate of the sacrum in the mid sagittal plane; a z-axis was set to be perpendicular to x-axis; a x-axis was set to be perpendicular to the x- and y-axes (Fig. 3) [14]. After labeling the sacrum and ilium for each posture, we registered the sacrum and calculated the position of ilium against sacrum. The angles were set as positive for anterior tilt in the x-axis, right bending in the y-axis and right rotation in the z-axis. As SIJ was fixed by glue, zero-rotation of all three planes from supine to standing position was considered the gold-standard (reference) value. The residual error was measured as the difference between the estimated and gold-standard rotations. Additionally, an experienced computer scientist in musculoskeletal image analysis, and an orthopedic surgeon traced the sacrum and ilium regions manually to assess the inter-rater variability in the tracing process on the registration accuracy. In addition, the orthopedic surgeon labeled these bones in a different time to evaluate intra-rater variability. We calculated x-rotation $\left( {\theta_{x} } \right)$, y-rotation $\left( {\theta_{y} } \right)$ and z-rotation $\left( {\theta_{z} } \right)$ for the ilium. The residual error between reference zero-rotation and the ilium rotation were calculated as follows

1 $$ {\text{Residual error}} = \sqrt {\theta_{x}^{2} + \theta_{y}^{2} + \theta_{z}^{2} } $$

The residual error was expressed as a mean value among three tests and the intra-rater and inter-rater variabilities were expressed as the difference of residual error between two tests.

### Kinematic study

Twenty healthy volunteers in their twenties were recruited for the study. One female was excluded because of blurred images by motion during the MRI scan. The remaining 19 volunteers (10 males, 9 females), with a mean age of 23.9 years (range, 22–29 years), were included. Those who were pregnant or had a past history of low back pain and lumber spine disorder were excluded. A 3D-upright MRI system was used for image acquisition. The 3D-MRI images of the left sacroiliac joint were acquired in both supine and standing positions using 3D HYCE with a 300 mm × 300 mm × 115 mm FOV.

**Fig. 4:**
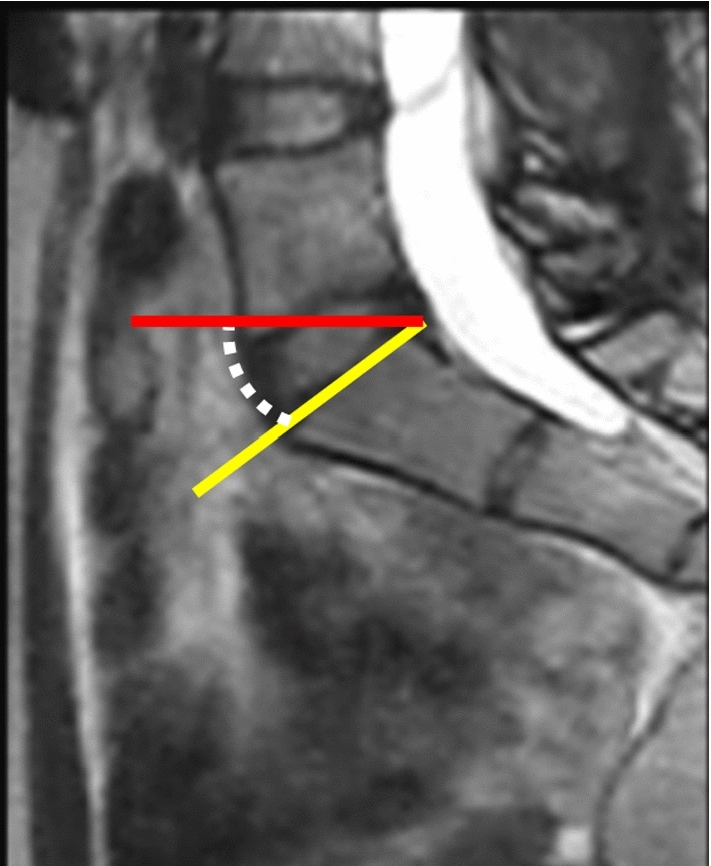
Measurement of sacral slope (SS). SS was defined as the angle between the superior endplate of S1 (yellow line) and vertical axis (red line) in the standing position, or horizontal axis in supine position

**Fig. 5:**
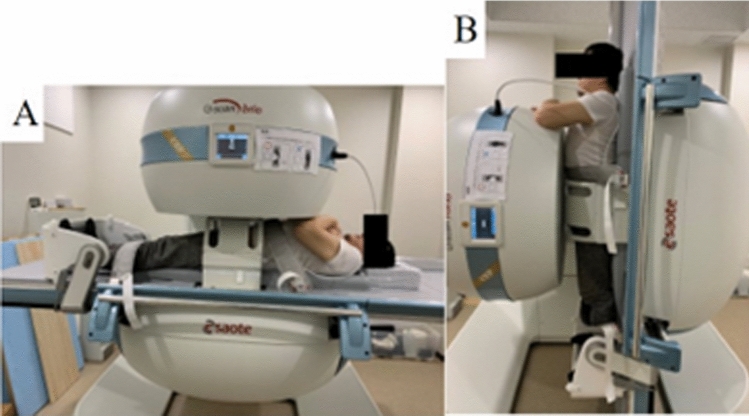
MRI was performed with the participant in the supine position **A** and the standing position **B**

An intensity-based registration method of sacrum and ilium between supine and standing MRI was performed to measure the rotation of the SIJ, as explained in Methods Sect. 1. The labels of the sacrum and ilium were traced manually in each position by an experienced computer scientist and verified by an orthopedic surgeon. For the sacral alignment parameter, sacral slope (SS) was measured in supine and standing positions. In supine position, SS is defined as the angle between the superior endplate of S1 and vertical axis [16]. In the standing position, SS was measured relative to the horizontal axis [17, 18] (Fig. 4). MRI measurements were evaluated with 3D viewer software (3D Template; Japan Medical Materials, Kyoto, Japan). Aforementioned reference coordinate system was used. Figure 5 shows the experimental setting for acquisition of the MR images in standing and supine positions.

### Statistical analysis

Sex differences in SIJ motion were evaluated using the Mann–Whitney *U*-test. The correlation between the sacral slope and kinematics of the SIJ was evaluated using the Pearson correlation coefficient. *P* values < 0.05 were considered a statistically significant difference. SPSS version 23 (IBM Japan, Tokyo, Japan) was used in the statistical analyses.

## Results

The static (reliability) study demonstrated that the residual errors of SIJ motion measurements were 0.17° in residual error, 0.02° in absolute x-rotation, 0.17° in absolute y-rotation, and 0.02° in absolute z-rotation. The inter-rater variability and intra-rater variability were 0.08° and 0.04°, respectively.

The means of SIJ motion in x-rotation from supine to standing position in male and female were − 0.9 ± 0.7° (mean ± standard deviation) and − 1.7 ± 0.8°, respectively (Table 1). A significant sex difference was seen in the x-rotation of the SIJ. Females showed a larger posterior rotation of the ilium against the sacrum in standing from the supine position than males (*p* = 0.04, Table 1). The x-rotation of the SIJ showed a significant correlation with the sacral slope in the supine and standing positions (r =  − 0.68, *p* = 0.001 and r = − 0.65, *p* = 0.002, respectively).

**Table 1:** Rotational motion of the sacroiliac joint against standing load

	Total	Female	Male	*P* value (female vs male)
*SIJ kinematics*				
x-rotation (°)	− 1.32 ± 0.8	− 1.7 ± 0.8	− 0.9 ± 0.7	0.04
y-rotation (°)	− 0.19 ± 0.43	− 0.06 ± 0.42	− 0.19 ± 0.45	0.74
z-rotation (°)	0.02 ± 0.32	0.01 ± 0.34	− 0.08 ± 0.33	0.32
*Sacral alignment*				
Sacral slope in supine (°)	35.6 ± 3.6	34.4 ± 3.6	37.0 ± 3.2	0.08
Sacral slope in standing (°)	31.7 ± 4.6	30.5 ± 4.0	33.0 ± 4.5	0.10
Change in sacral slope from supine to standing (°)	− 3.9 ± 3.1	− 3.9 ± 2.2	− 3.8 ± 3.5	0.71

Angles expressed as mean ± standard deviation

*SIJ* Sacroiliac joint

## Discussion

We used the upright 3D MRI system to evaluate the kinematics of the SIJ against standing load. The residual error of measurement of SIJ motion was < 0.2°. Thus, our method was considered sufficiently accurate to detect > 0.5° of SIJ motion against standing load. To the best of our knowledge, this study is the first to investigate the SIJ motion against standing load in healthy young adults using MRI. Our results showed that changes in the SIJ from the supine position to standing position were 0.9° in male and 1.7° in female. The postural change in the sacroiliac joint in female rotated more posteriorly than that in male.

The literature on the biomechanical differences between the male and female SIJ is limited, although significant sex differences in pelvic ring morphology exist [1]. Joukar A et al. developed the validated finite element models of a male and a female lumbar spine-pelvis-femur from CT scans [12]. They reported that the female SIJ had higher mobility in compressive load and bending moment than the male SIJ. Similarly, this study showed that female had higher SIJ mobility in standing load than men. Our result may support these findings from a simulation study of SIJ motion.

This study showed a significant correlation between the sacral slope in the standing position and sagittal rotation of the SIJ. A high sacral slope in standing means a ventral shift of the gravity line relative to the SIJ. An increased lever arm in the sagittal plane may cause higher posterior rotation of the SIJ. However, there were no significant sex differences in the sacral slope, which indicated that the sacral slope is not the cause of sex differences in SIJ motion. A 3D analysis of spino-pelvic alignment in 60 asymptomatic young adult males and females reported that the female spine was more dorsally inclined, evaluating T1–L5 sagittal spinal inclination, which is the angle between the vertical line and a best fit straight line passing through the center of vertebrae (T1–L5). And there were no significant sex differences in the sacral slope [19]. Moreover, males have been shown to have a more rigid SIJ mobility than females [12, 20]. Our results support these studies. Several reports have indicated that pregnancy is likely to result in SIJ pain and asymmetric SIJ laxity [6, 21–23]. However, we did not include pregnant females in our study. Thus, this sex difference in SIJ motion may explain why SIJ pain and low back pain are more common in females than in males. A further kinematic study is needed in the patients with SIJ pain to elucidate the pathology of SIJ pain using the upright MRI.

There are some limitations to this study. First, the results may be subject to bias because we only included volunteers in their twenties. A previous study demonstrated that patients with degenerative lumbar disorder have higher mobility of the SIJ than healthy adults [14]. Thus, aging and disorders of the spine or hip may change the kinematics of the SIJ. Therefore, a further study is needed to elucidate the effect of age and adjacent joint disorders on the kinematics of the SIJ. Second, we manually traced the sacrum and ilium in the supine and standing positions, which may have affected the residual error of measurement. However, in this study, the residual error of SIJ motion measurements was < 0.2° in all rotations. Thus, our method is sufficiently accurate because there was 0.8° of SIJ motion difference between male and female against standing load. The upright MRI is important for clarifying SIJ pain and low back pain, and enables the investigation of SIJ motion in males and females without the use of radiation. Therefore, additional system development to allow for automatic tracing of the ilium and sacrum is needed to increase the number of participants in a future study. Third, a fixed dry pelvic ring bone and gel foam were used in static (reliability) study to make the environment similar to bone in vivo. These materials may affect static (reliability) study. As it is impossible to make an environment in vivo (the amount of water in the human body is approximately 70% [24]), gel foam was used instead of water. Additionally, we used dry bone and not plastic bone, which is likely to have a minimal effect on the static (reliability) study. We used in only one dry bone in static (reliability) study. Thus, the effect of morphological variations on the measurement accuracy could not be evaluated. The inter-rater variability and intra-rater variability were 0.08° and 0.04°, respectively, which was sufficient for the target analysis. Finally, our study showed that young healthy participants had a sex difference in the SIJ mobility but the difference was small (0.8°). A previous FEM study indicated that female SIJ had higher mobility, stresses, loads, and pelvis ligament strains compared with the male SIJ which led to higher stress across the joint, although the gender difference of SIJ rotation was small (0.3°–0.9°). Our findings align with the EEM study and even a small difference in SIJ motion might be a possible reason for higher incidence of SIJ pain and pelvic stress fracture in females. [12]. Additional investigation on the patients with low back pain would be necessary to elucidate the mechanism of low back pain associated with SIJ.

## Conclusion

The residual error of measurement of SIJ motion using the upright MRI was < 0.2° in all rotations. Young healthy participants showed a sex difference in antero-posterior rotation of the SIJ against the standing load, and females showed a more posterior rotation of the ilium against the sacrum from the supine to standing position than males. The SIJ motion difference between males and females was 0.8°, and upright MRI was useful to investigate SIJ motion.
